# Quality of health care in adolescents and adults with disorders/differences of sex development (DSD) in six European countries (dsd-LIFE)

**DOI:** 10.1186/s12913-018-3342-0

**Published:** 2018-07-05

**Authors:** Ute Thyen, Till Ittermann, Steffen Flessa, Holger Muehlan, Wiebke Birnbaum, Marion Rapp, Louise Marshall, Maria Szarras-Capnik, Claire Bouvattier, Baudewijntje P. C. Kreukels, Anna Nordenstroem, Robert Roehle, Birgit Koehler, on behalf of the dsd-LIFE group, Birgit Koehler, Peggy Cohen-Kettenis, Annelou de Vries, Wiebke Arlt, Claudia Wiesemann, Jolanta Slowikowska-Hilczer, Aude Brac de la Perriere, Charles Sultan, Francoise Paris, Claire Bouvattier, Ute Thyen, Nicole Reisch, Annette Richter-Unruh, Hedi Claahsen-van der Grinten, Anna Nordenstrom, Catherine Pienkowski, Maria Szarras-Czapnik

**Affiliations:** 1 Klinik fur Kinder- und Jugendmedizin, Universitat zu Lubeck, Ratzeburger Allee 160, 23538 Lubeck, Germany; 2 Institute for Community Medicine – SHIP-KEF, Ernst-Moritz-Arndt University Greifswald, Walther-Rathenau-Str. 48, 17487 Greifswald, Germany; 3 Department of Health Care Management, University of Greifswald, 17487 Greifswald, Germany; 4 Institute of Psychology, Department Health & Prevention, Ernst-Moritz-Arndt University Greifswald, Robert-Blum-Str. 13, 17487 Greifswald, Germany; 5 Klinik fur Kinder- und Jugendmedizin, Sektion Padiatrische Endokrinologie, Universitat zu Lubeck, Ratzeburger Allee 160, 23538 Lubeck, Germany; 6 Department of Endocrinology and Diabetology, Children’s Memorial Health Institute, Warsaw, Poland; 7 Endocrinologie pediatrique, Centre de reference des maladies rares du developpement sexuel, Hopital Bicetre, Universite Paris-Sud, 78 rue du General Leclerc, 94270 Paris, Le Kremlin Bicetre France; 8 Medische Psychologie en Medisch Maatschappelijk Werk, VU Medisch Centrum, PO Box 7057, 1007 MB Amsterdam, the Netherlands; 9 Department of Women’s and Children’s Health, Karolinska Institutet, Karolinska University Hospital, 171 76 Stockholm, Sweden; 10 Koordinierungszentrum fur Klinische Studien (KKS) Charité - Universitätsmedizin Berlin, Corporate Member of Freie Universitat Berlin, Humboldt-Universität zu Berlin, Berlin, Germany; 11 Klinik für Padiatrische Endokrinologie und Diabetologie, Charité - Universitätsmedizin Berlin, Corporate Member of Freie Universität Berlin, Humboldt-Universität zu Berlin, Berlin, Germany

**Keywords:** Disorders/differences of sex development, Patient satisfaction, Multidisciplinary care

## Abstract

**Background:**

To investigate the association between the structural quality of care and patient satisfaction with care in individuals with disorders/ differences of sex development (DSD).

**Methods:**

A multicenter cross-sectional comparative study was conducted in 14 clinics in six European countries. We assessed the level of structural quality of care in each center using a self-constructed measure (Center Score) and the level of participant satisfaction with care using the customer satisfaction questionnaire (CSQ-4) and an adopted version of the Youth Health Care – Satisfaction, Utilization & Needs (YHC-SUN-SF). Data were obtained from individuals with Turner Syndrome (261), Klinefelter Syndrome (173), 46, XX congenital adrenal hyperplasia (190) and XY-DSD (257).

**Results:**

We found large variations between the scores for structural quality of care both within a diagnostic group and within a country; the overall association between participant satisfaction with the center score was significant.

**Conclusions:**

Comparative effectiveness research across Europe can lead to more insight on beneficial structures and processes and the overall strategy to care for people with rare diseases in general and specific conditions such as disorders/ differences of sex development. Appreciation of higher levels of structural quality of the centers in this study supports the concept of comprehensive care.

**Trial registration:**

German Clinical Trials Register: Registration identification number: DRKS00006072, date of registration April 17th, 2014.

DRKS00006072 (German Clinical Trials Register).

## Background

Rare diseases are typified by a lifetime prevalence and an incidence of less than 5 per 10,000 individuals. Approximately 80% of rare diseases have a genetic background, and 50% manifest in early childhood. In the latter case, the diseases are often associated with high mortality. Affected individuals can be identified early through neonatal screening programs when available (i.e., metabolic disease, hormonal deficiencies such as hypothyroidism or congenital adrenal hyperplasia). Others often require years to achieve an established diagnosis. Structured and competent care sites are often missing, and quality-checked information for affected patients along with evidence-based guidelines for clinicians are often lacking [1]. While the basic science of these diseases is often well-established [2], translation of this knowledge into a clinical benefit for patients has been slow, and deficits in specialized care or lack of access to such care create difficulties with diagnosis and service provision. Because of the low prevalence and geographic distribution of patients and researchers, both the research and organization of care on rare diseases suffer from infrastructural deficits [3].

In Europe, EURORDIS, an umbrella organization of patient associations in rare diseases, has been supporting the work of the European commission since the early 2000s to improve care for people with rare diseases. Joint efforts on the EU level to build Reference Networks for various diagnostic groups (ERN-European Reference Networks for Rare Conditions) have been the latest cornerstone in improving access to high-quality care and establishing standards in diagnostic care, including the development of quality and safety benchmarks [4–6].

The dsd-LIFE consortium sought to address these issues in a multicenter European clinical study on the outcome of surgical and hormonal therapy and psychological intervention in disorders/ differences of sex development (www.dsd-LIFE.eu) as an example of many problems in health care in rare diseases. Disorders/ differences of sex development (DSD) are defined as congenital conditions in which the development of chromosomal, gonadal and/or anatomic sex is atypical, following the statement of the Chicago Consensus Meeting in 2005 [7]. DSD comprises sex chromosome conditions (including Turner Syndrome (TS), Klinefelter syndrome (KS) and mixed gonadal dysgenesis (GD)), conditions with a 46, XY karyotype including complete/partial androgen insensitivity syndrome (AIS), complete/partial GD, steroid synthesis deficiencies and severe hypospadias (> = grade II) and conditions with a 46, XX karyotype (including Congenital Adrenal Hyperplasia (CAH) in females, GD and XX men). The overall incidence has been estimated at approximately 1 per 4500–5000 people but varies considerably between specific diagnoses at 1:500–1000 for KS, 1:2500 for TS, 1:15,000 in CAH and 1:150,000 for androgen synthesis deficiencies.

Management of DSD conditions is challenging because people with DSD face complex medical and psychosocial hurdles, including hormone treatments, cancer risk, gender identity, fertility and sexuality [8, 9]. Further health problems and chronic physical and mental diseases that are both related and unrelated to the specific DSD diagnosis must be considered [10]. Therefore, as a major outcome of the Chicago Consensus Conference, multidisciplinary care is recommended and has been re-emphasized in an updated recommendation of the Chicago Consensus in 2016 [11]. Centers of reference should implement a working team that includes specialists in endocrinology, surgery and/or urology, clinical psychology/psychiatry, pathology and gynecology as the minimum standard [12, 13].

This complex process has also been supported by the recommendations of national, ethical stakeholders [14, 15]. National Centers of Expertise and National Networks have been established in many European Countries in recent years [6]. However, quality indicators have not been standardized, and the criteria to define a center of reference or a center of excellence vary. Therefore we designed an exploratory study to investigate the impact of aspects of structural quality of care including aspects of infrastructure on the hospital and clinic level and service delivery on patient satisfaction with care.

The term “quality” is crucial for this analysis but there is no generally accepted definition. In this paper, we follow the quality model of Donabedian [16–18] as it was developed specifically for the health care sector and is widely used in health care management [19]. Donabedian’s model follows the production system of transforming inputs into outputs in a multi-stage process. Structures (e.g. equipment and buildings, materials, labor) are transformed in absence of the patient into a stand-by-capacity. Finally, the stand-by-capacity and further agents of production are combined in presence of the patient to an output. In the health care setting, this output is usually a service which induces a certain outcome for the patient and an impact for the society. Consequently, the three stages (structure, process, output) can be analysed from a quantitative (quantities of inputs, quantities flowing through the process, quantities of outputs) and qualitative (quality of inputs, quality of processes, quality of results) perspective. Donabedian calls the first perspective “structural quality”, the second perspective “process quality” and the last perspective “results quality”. Typical elements of the structural quality are the qualification of personnel, innovativeness of equipment and buildings, space of buildings, accessibility etc. Process quality is reflected by the adequacy of internal production processes, such as waiting times, documentation and sequencing. The dimensions of the result quality are the subjective and objective qualities of the services rendered to the patient.

Donabedian is well-known for his conditional chain between the elements of the quality production. He assumes that a good structural quality is a necessary, but not sufficient condition of good process quality, whereas good process quality is a necessary, but not sufficient condition of good result quality. Thus, good structural quality is a prerequisite of good result quality but not a guarantee. Following the production model we may assume that a good structural quality is indeed a good proxy for the objective result quality but not for the satisfaction of the patient (subjective result quality), i.e., a proper quality assessment will require an analysis of structural quality and subjective result quality.

The model of Donabedian is more detailed than the well-known “structure-process-result” sequence. He also distinguishes the dimensions accessibility, organization, physician-patient-relationship and continuity.

In this study, we collected data for the structural quality of the components of care relevant for services for patients with rare conditions of the centers that recruited the participants to the study. We designed a measure to assess the structural quality according to Donabedian, including the infrastructure of care on the hospital and clinic level and the quality such infrastructure including components of service delivery. For the satisfaction with care from the participants’ perspectives (subjective result quality according to Donabedian) we collected data from the participating individuals receiving care in the centers.

Thus, we neglect the process quality assuming that a good subjective result quality and a good structural quality are only possible if the process quality is good as well. This follows the structure-process-results paradigm of Donabedian.

For the framework to design the measure we followed the information available from the literature related to the European Reference Networks [4, 6, 10, 15], in Germany represented by the National Action League for People with Rare Diseases (NAMSE) which has published criteria to define the level of expertise and comprehensiveness of care for national centers of expertise [20]. Central to the concept is a minimum number of patients seen per year allowing increasing experience, maintaining a registry, and participating in clinical studies. Access to specialized services includes the availability of molecular genetic testing crucial for making a diagnosis in this population. In terms of clinical services a multiprofessional care team including various medical specialists and other professionals is mandatory. Larger teams indicate more variety and availability of professions and patients consult with team members according to their needs. Following the team approach regular case conferences and individual case management are required features for good service delivery; additional features include close collaboration with patient support groups and providing health information as well as organization of transitional care for adolescents and good availability of expertise between clinic visits (i.e., telephone counselling) [4, 6, 10, 20].

This cross-sectional clinical evaluation study, dsd-LIFE, is the first large multi-center European study of people with DSD to date. The objectives of this paper are as follows:

1. To explore variations of care for four types of DSD,
2. To describe satisfaction with health care services,
3. To assess the association between structural quality of care and satisfaction with care.

## Methods

### Study design

The methods of the multicenter cross-sectional clinical evaluation study, dsd-LIFE, are described in detail elsewhere [21] and are summarized below. The dsd-LIFE consortium consisted of 16 European partners from Germany, France, the Netherlands, Poland, Sweden and the United Kingdom (UK), of whom fourteen were active recruiting sites. Recruitment of adolescents (≥ 16 years) and adults with DSD occurred from February 2014 to September 2015. A total of 3100 eligible people were approached, of whom 1040 participated in the study. All individuals met the inclusion criteria for having a DSD condition as described in the classification system of the Chicago Consensus Conference [7].

The dsd-LIFE assessment consisted of two parts. The first part included a medical interview, a retrospective chart review and medical examinations; all were carried out by trained researchers following standard operation procedures. The second part of the study consisted of a patient-reported outcome questionnaire (PROQ). The PROQ was administered as an online version and was accessible only with a secure password at the recruitment centers. If needed, a paper-pencil version was provided. The PROQ included sociodemographic data (including age, education, residence, and relationship status), contemporary health status and satisfaction with health care.

### Instruments and measurements

Scoring instrument for structural quality of centers (*Center score*): We developed the assessment of the extent of multidisciplinary care provided in the 14 centers by probing 17 items per the four diagnostic groups (TS, KS, CAH and XY-DSD). In each of the 14 participating centers the clinical representative for the dsd-LIFE consortium documented all information requested in March 2017 to represent the structural quality of the centers at the time of the recruitment period in 2015. Three items did not have discriminatory power and were excluded from the scoring system. The deleted items asked whether the center served all age groups, provided expert advice / second opinions and was involved in teaching and training activities. The response to these items showed no variation with very high ceiling effects. The remaining 14 items were assigned to different structural domains of health care.

1. *Infrastructure at the hospital level (6 items)*:

- size of the center (response scale: number of patients per year;
- scoring below the median of all centers = 0, above the median = 1);
- acknowledgment as a center of reference (scoring: no = 0; yes = 1);
- participation in registries (scoring: no = 0; yes = 1);
- national or international collaborations excluding dsd-LIFE (scoring: no = 0; yes = 1);
- Participation in clinical trials (scoring: no = 0; yes = 1);
- Access to molecular genetic testing (scoring: no = 0; yes = 1);

2. *Infrastructure at the clinic level (2 items)*

- team-size (response scale: number of different professions represented in the team calculated from the positive items of a list of professions: geneticist, endocrinologist, internal medicine subspecialist (other than endocrinologist), gynaecologist, urologist, surgeon (other than gynaecologist and urologist), psychiatrist, psychologist, social worker, casemanager, and other professions; scoring: below the median of all centers = 0, above the median = 1);
- options for referrals (scoring: no = 0; yes = 1),

3. *Service delivery (6 items)*:

- availability of case management (scoring: no = 0; yes = 1),
- transitional care (scoring: no = 0; yes = 1),
- collaboration with patient organizations (scoring: no = 0; yes = 1),
- conduct of case conferences (scoring: no = 0; yes = 1),
- access to educational materials for patients (scoring: no = 0; yes = 1),
- telephone counseling (scoring: no = 0; yes = 1).

We added up the scores across the 14 items and calculated a mean score for each clinic across the 14 centers yielding 56 different scores ranging theoretically from 0 (min) to 14 (max) and in fact from 2 to 10. Subsequently we added the score to each participant’s individual data in the data set according to their respective center.

### Satisfaction with health care

Satisfaction with health care was measured on a general level by the Customer Satisfaction Questionnaire (CSQ-4) [22] and on a domain-specific level with a modified short-form of the Youth Health Care – Satisfaction, Utilization and Needs (YHC-SUN-SF) measure [23].

#### Customer satisfaction questionnaire (CSQ-4)

The CSQ-4 is a self-report questionnaire constructed to measure satisfaction with services in general [22]. An original item pool of 81 statements was sequentially reduced to establish the CSQ-8 with 8 items [24, 25] and the subsequent CSQ-3 with items 3, 7, and 8 from the CSQ-8. In the present study, the CSQ-4 was applied, which comprises the CSQ-3 item set and includes an additional item measuring improvement in self-efficacy: “Have the services helped you deal more effectively with your problems?” The verbal anchors of the response choice options differ from item to item but are all based on a four-point Likert scale without a neutral position. An overall score of the single dimension is produced by the unweighted summation of the direction-corrected response values, ranging from 4 (“most dissatisfied”) to 16 (“most satisfied”) [26]. Questions were slightly adapted to dsd-LIFE as recommended by the developers of the questionnaire to access general satisfaction across various health and human services, e.g., from “To what extent has our program met your needs?” to “To what extent has the treatment you received met your needs?”. For participants with at least one missing answer, the calculation of the CSQ-4 was not possible and the response was coded as invalid.

#### Youth health care – Satisfaction, utilization & needs short-form (YHC-SUN-SF)

The self-reported measure YHC-SUN-SF was developed after the cross-culturally parent-reported “Child Health Care – Satisfaction, Utilization and Needs” (CHC-SUN) showed good psychometric properties and appropriate clinical use for the evaluation of pediatric health care services for children with special health care needs (CHC-SUN) from the proxy perspective of parents [27, 28]. YHC-SUN-SF comprises two modules: a module covering “provision of health care services” and a module covering “satisfaction with health care”. Later, a self-reported version for adolescents “The Youth Health care measure - Satisfaction, utilization, and needs (YHC-SUN)” was developed [23] as well as short-forms of both versions (CHC−/ YHC-SUN-SF).

The YHC-SUN-SF consists of 30 single items, which were also assigned to two modules of which only data assessed with module 2 were used in this study. The items in module 2 were assigned to the domains “diagnosis/information”, “patient-centered care”, and “doctors’ behavior”. The single item on “general satisfaction with health care” was not included in this section because the same question was already part of the CSQ-4 (but the answer structure was a four-point instead of five-point-Likert scale). For module 2, the response choices on a five-point scale were “not satisfied”, “partly satisfied”, “satisfied”, “very satisfied”, and “extremely satisfied”. The time frame comprises the previous 12 months. All questions of the YHC-SUN short-form used in the German evaluation study of a modular training approach (ModuS) for chronically ill adolescents, aged 15 to 24 years, were included in the PROQ of dsd-LIFE. The adopted measure with 10 items was analyzed in terms of psychometric performance of this extended short-form. The results lend support to the structural assumptions relating to the adopted YHC-SUN-SF. The model with the three factors “diagnosis/information”, “patient-centered care” and “doctors’ behavior” better fits the empirical data than a model with a single general factor. Moreover, the German, French, Dutch and Swedish versions of the YHC-SUN-SF exhibit measurement invariance (i.e., samples from UK and Poland had been excluded from these analyses due to small sample sizes).

### Sociodemographics

Participants were classified as male, female, or other than male or female, based on how they identified in the medical interview. The educational level was reported per the ESISCED, a European standardized education measurement from the European Social Survey (www.europeansocialsurvey.org/, round 6, 2012), and levels 1 and 2 were categorized as low education, levels 3 to 5 as medium education and levels 6 and 7 as high education. Economic status was evaluated with a question addressing feelings about household income with “living comfortably on present income”, “coping on present income” and “finding it (very) difficult on present income” as answer categories (www.europeansocialsurvey.org/, round 6, 2012).

#### General health

Contemporary health status was measured by the general questions “How is your health in general? Would you say it is (very) bad to (very) good?” and the question “Do you have any longstanding illness or health problem?” focusing on physical, mental or mixed conditions in the answers (www.europeansocialsurvey.org/, round 6, 2012).

### Statistics

For the following analyses, we grouped participants into five categories: female TS, male KS (including male XYY with gonadal dysgenesis), female CAH, female XY-DSD and male XY-DSD. Male and female XY-DSD groups comprised the diagnosis: (complete/partial/mixed) GD with all types of karyotype, complete/partial AIS, androgen synthesis defects, severe hypospadias and other rare diagnoses not included into female TS, male KS and female CAH. Participants identifying as “other than male or female” or “not the typical gender” for the condition were excluded from the diagnosis-specific analyses (*n* = 18). This was necessary due to a high co-linearity between diagnosis group and gender. Stratified by patient group, continuous variables are described by median and the 25th and 75th percentiles, and continuous variables are presented as absolute numbers and percentages. Coefficients for inter-item correlations were calculated via the Somers‘ D statistic (Somers’ *D* takes values between – 1 when all pairs of the variables disagree and 1 when all pairs of the variables agree). Correlations between single items and total score of the center score were assessed by ANOVA.

Associations of the center score with the CSQ-4 and YHC-SUN-SF were analyzed using linear regression models for all patients together – one unadjusted analysis and one analysis adjusted for age, general health and country. The results are expressed as the β coefficient, 95% confidence interval and *p*-value. A *p* <  0.05 was considered statistically significant. Furthermore, interactions of the center score with the countries were tested in the CSQ-4 model. Here, a *p* <  0.1 was considered statistically significant. The results of the interaction model are displayed as country-specific regression lines for the center score on the CSQ-4. All analyses were carried out with Stata 14.1 (Stata Corporation, College Station, TX, USA).

## Results

### Recruitment

We included 948 participants with valid answers to the CSQ-4 into the analysis. In total, 279 had TS, 189 had KS, 211 had CAH, 184 females had XY-DSD and 85 males had XY-DSD. The recruitment strategies and sizes of the corresponding clinics among the participating centers showed large variations, and therefore, the numbers of diagnostic groups were unequally distributed among the centers. The highest number of participants with CAH originated from Germany, followed by KS from the Netherlands and TS from France, whereas participants with XY DSD originated from all countries albeit with small numbers. The largest sample with the rarest conditions (males with XY-DSD) originated from Poland (Table 1).

**Table 1:** Participation across centers (n, %)

Country	Number of centers	Turner syndrome (TS)	Klinefelter syndrome (KS)	Congenital adrenal hyperplasia (CAH)	Female XY-DSD	Male XY-DSD	Total
Germany	Four	43 (15.41)	33 (17.46)	88 (41.71)	48 (26.09)	18 (21.18)	230 (24.26)
France	Four	102 (36.56)	25 (13.23)	58 (27.49)	47 (25.54)	12 (14.12)	244 (25.74)
Netherlands	Two	74 (26.52)	68 (35.98)	22 (10.43)	44 (23.91)	2 (2.35)	210 (22.15)
Poland	Two	3 (1.08)	22 (11.64)	14 (6.64)	27 (14.67)	39 (45.88)	105 (11.08)
Sweden	One	46 (16.49)	33 (17.46)	11 (5.21)	15 (8.15)	12 (14.12)	117 (12.34)
United Kingdom	One	11 (3.94)	8 (4.23)	18 (8.53)	3 (1.63)	2 (2.35)	42 (4.43)
Total	14	279 (100)	189 (100)	211 (100)	184 (100)	85 (100)	948 (100)

Total Pearson chi2(4) = 948.0000 Pr = 0.000

### Sociodemographics and general health

Participants with KS were significantly older, less likely to achieve high educational levels and least likely to live comfortably in their social contexts compared to other people. Participants with XY-DSD were younger. Female patients with XY-DSD, in particular, reported significantly higher educational levels. We did not find significant differences in feelings about household income (Table 2).

**Table 2:** Sociodemographic data, health status and satisfaction with care of the study participants responding to the CSQ-4 (*n* = 948; %)

Variable	Categories	Turner syndrome (TS)	Klinefelter syndrome (KS)	Congenital adrenal hyperplasia (CAH)	Female XY-DSD	Male XY-DSD	Total
Age	p50 [p25; p75]	29 [21; 43]	37 [27; 51]	29 [21, 38]	26.5 [21; 36.5]	22 [18; 29]	29 [21; 41]
Education	Low	40 (15.33)	53 (30.64)	40 (21.05)	29 (16.67)	26 (31.33)	188 (21.34)
	Medium	130 (49.81)	94 (54.34)	100 (52.63)	76 (43.68)	36 (43.37)	436 (49.49)
	High	91 (34.87)	26 (15.03)	50 (26.32)	69 (39.66)	21 (25.30)	257 (29.17)
Feeling about household income	Finding it (very) difficult to live on present income	35 (14.29)	29 (17.26)	30 (16.22)	19 (11.11)	8 (14.04)	121 (14.65)
	Coping with present income	113 (46.12)	83 (49.40)	78 (42.16)	73 (42.69)	31 (54.39)	378 (45.76)
	Living comfortably on present income	97 (39.59)	56 (33.33)	77 (41.62)	79 (46.20)	18 (31.58)	327 (39.59)
Health status	Very bad	1 (0.36)	4 (1.9)	4 (1.9)	3 (1.63)	0 (0)	12 (1.27)
	Bad	8 (2.87)	22 (11.64)	11 (5.21)	11 (5.98)	13 (15.29)	65 (6.86)
	Fair	80 (28.67)	61 (32.28)	62 (29.38)	45 (24.46)	23 (27.06)	271 (28.59)
	Good	167 (59.86)	85 (44.97)	102 (48.34)	90 (48.91)	36 (42.35)	480 (50.63)
	Very good	23 (8.24)	17 (8.99)	32 (15.17)	35 (19.02)	13 (15.29)	120 (12.66)
Satisfaction with care	CSQ4 p50 [p25; p75]	14 (12; 15)	13 (11; 15)	14 (12; 15)	13 (11; 15)	13 (11; 15)	13 (12; 15)
	YHC-SF total satisfaction	56 (47; 75)	53 (42; 75)	61 (50; 83)	58 (42; 81)	53 (47; 75)	56 (47; 75)
	YHC-SF info satisfaction	50 (50; 75)	50 (33; 75)	58 (50; 75)	50 (38; 75)	50 (33; 75)	50 (42; 75)
	YHC-SF care satisfaction	50 (50; 75)	50 (42; 75)	58 (50; 83)	58 (42; 83)	50 (50; 75)	50 (50; 75)
	YHC-SF doctor satisfaction	58 (50; 75)	58 (50; 75)	75 (50; 92)	67 (50; 83)	67 (50; 83)	67 (50; 83)

Differences between diagnostic groups: age (Test for difference in median) *p* = 0.000; education (Pearson chi2 (8) = 42.3919), Pr = 0.000; income (Pearson chi2(8) = 9.5527), Pr = 0.298; general health (Pearson chi2(16) = 50.6480) Pr = 0.000; global satisfaction with care CSQ4 (Test for difference in median) *p* = 0.022; YHC-SF total satisfaction *p* = 0.209; YHC-SF info satisfaction *p* = 0.189; YHC-SF care satisfaction *p* = 0.049; YHC-SF doctor satisfaction *p* = 0.265

Participants with KS and male patients with XY-DSD were most likely to report bad or very bad general health (Table 2).

### Center scores

Except a few coefficients we primarily found low to moderate correlations among the 14 individual items and with the total score. The correlations do not suggest a dimensional structure and we use it as a unidimensional measure called center score (Table 3).

**Table 3:** Correlations among single items of the measure of structural quality and with total score

	CS	RC	Reg	Col	CT	MG	TS	Ref	CM	TC	PO	CC	EM	PC
Total score	0.34	0.47	0.55	0.17	0.59	0.37	0.36	0.21	0.18	0.16	0.57	0.18	0.17	0.29
Infrastructure at hospital level														
Clinic Size (CS)		0.24	0.37	0.29	0.33	0.31	0.36	0.26	0.16	0.16	0.48	0.09	0.16	0.06
Reference Center (RC)			0.69	0.27	0.52	0.19	0.43	0.52	0.12	0.19	0.46	0.27	0.29	0.76
Registry (Reg)				0.28	0.74	0.36	0.54	0.26	0.24	0.22	0.50	0.19	0.26	0.33
Collaboration (Col)					0.33	0.03	0.20	0.11	0.08	0.08	0.27	0.14	0.08	0.19
Clincal Trials (CT)						0.29	0.57	0.22	0.26	0.47	0.55	0.18	0.29	0.42
Molecular Genetics (MG)							0.27	0.14	0.19	0.24	0.65	0.18	0.42	0.00
Infrastructure at the clinic level														
Teamsize (TS)								0.04	0.04	0.05	0.35	0.31	0.21	0.41
Referrals (Ref)									0.37	0.09	0.22	0.04	0.07	0.26
Service delivery														
Casemanager (CM)										0.17	0.23	0.36	0.17	0.19
Transition Care (TC)											0.27	0.01	0.05	0.37
Patient Organization (PO)												0.17	0.65	0.17
Case Conferences (CC)													0.02	0.25
Education Material (EM)														0.09
Phone Counseling (PC)														

We found large variations between the center scores both within a diagnostic group and within countries, with differences exceeding 10 score points (given a theoretical score range from 0 to 14 points) (Table 4).

**Table 4:** Variation among center scores by country and per diagnostic group

Country	Number of centers	Turner syndrome (TS)	Klinefelter syndrome (KS)	Congenital adrenal hyperplasia (CAH)	XY-DSD (female and male)
Germany	Four	1–12	2–10	10–12	1–12
France	Four	9–14	10–12	9–12	10–12
Netherlands	Two	8–12	5–7	3–13	8–12
Poland	Two	1–5	2–4	0–4	3–4
Sweden	One	12	9	10	6
United Kingdom	One	8	5	12	10
Minimal-maximal score		1–14	2–12	0–13	1–12
Median [P25, P75])		11 [7; 12]	7 [5; 9]	12 [10; 12]	6 [3;10]

Test for difference in median c_score *p* = 0.000

Variations within centers for different diagnostic groups/clinics were generally smaller. In Germany and its four centers, we found the largest variations both within diagnostic groups and within centers. Overall scores ranged from 1 to 12; ranges within the four German centers were 4–6 score points different (data not shown) except for one center in Germany with high scores (10) for CAH and very low scores for the other 3 diagnostic groups (1–2). Variations within the French centers were small, ranging from a score of 9 to a maximum score of 14 across all four centers. The four centers generally had the same scores for each diagnostic group with differences of 2–4 score points within a center. The two centers in the Netherlands were quite different with scores ranging from 3 to 12 in one center and 7–13 in the second center for the four conditions. One center appeared to be more specialized in CAH with a high score in that condition (score of 13), whereas the other center reported the highest score for XY-DSD (score of 12). The two centers in Poland reported scores between 0 and 3 in one center and 4 and 5 in the other center. The Swedish center reported a center score of 10 for CAH and a somewhat lower score for KS (score of 9), TS (7) and XY-DSD (score of 6). The UK center reported scores of ≥10 for CAH and XY-DSD and lower scores for TS (score of 8) and KS (score of 5).

At the individual level, we found a large array across all possible center scores, ranging from 35 (3.7%) individuals recruited in a clinic with a score of 0–1, 87 (9.2%) individuals in a clinic with a score of 2–3, 120 (12.7%) individuals in clinics with a score of 4–6 and 253 (26.6%) in clinics with a score of 7–9 [data not shown]. Thus, approximately half of the patients included in the study received care or had access to clinics with center scores of 10 points or higher. Participants with CAH were most likely to be cared for in centers with high scores followed by participants with TS. Participants with KS and those with XY DSD were less likely to be cared for in centers with high scores for multidisciplinary care. In the latter group, the scores were lower for male XY-DSD participants (median 4, p25 3, p75 7) compared to female XY-DSD participants (median 7, p25 4, p75 11) (Table 4).

### Satisfaction with care

Global satisfaction with care (CSQ-4) showed marginal differences with slightly higher levels of satisfaction in CAH and TS compared to the other diagnostic groups (*p* < 0.05). There were no differences between the total score and three subscales of the YHC-SUN-SF except for the subscale “patient-centered care” (*p* < 0.05) (Table 2), with higher values for CAH and female XY-DSD.

### Association between structural quality of care (center score) and satisfaction with care (Table 5, Fig. 1)

**Fig. 1:**
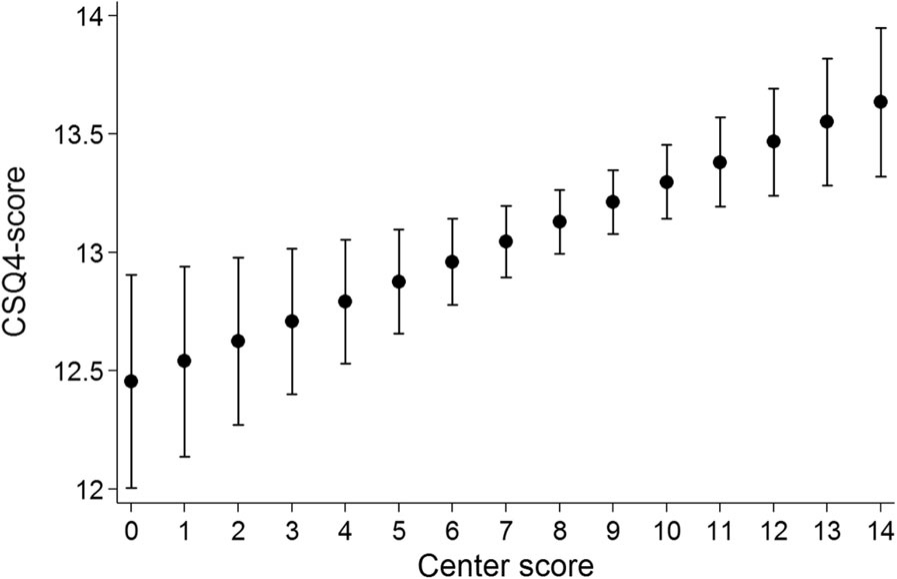
Association of the center score with satisfaction with care

Overall association of the center score for structural quality of care with individual participant satisfaction with care was significant with *p* < 0.001. As shown in Fig. 1, over the full-scale range of the possible center score values, the average satisfaction score only varied approximately 1 point between 12.5 (given a minimum center score) and 13.5 (given a maximum center score), indicating a substantial yet limited association between structural components and subjective evaluation of health care. The unadjusted regression of general satisfaction with care (CSQ-4 score) and the center score explained only 3% of the variance. However, after adjusting for age, health status and country, 16.6% of the variance could be explained. We did not find a significant association between center score and diagnostic groups. However, a single interaction effect (*p* < 0.05) between the center score and general satisfaction with care (CSQ-4 score) for a diagnostic group (male XY-DSD) was significant.

**Table 5:** Association between center score and satisfaction with care

	CSQ-4	YHC-SF total satisfaction	YHC-SF info satisfaction	YHC-SF care satisfaction	YHC-SF doctor satisfaction
Center score	0.09 (0.04; 0.14); *p* < 0.001	0.64 (0.11; 1.17); *p* = 0.019	0.44 (−0.13; 1.01); *p* = 0.130	0.64 (0.06; 1.21); *p* = 0.031	0.83 (0.27; 1.40); *p* = 0.004
Age; years	−0.01 (−0.02; 0.00); *p* = 0.143	−0.10 (−0.22; 0.02); *p* = 0.111	−0.03 (−0.16; 0.10); *p* = 0.647	− 0.12 (− 0.26; 0.01); *p* = 0.065	−0.15 (− 0.28; − 0.02); *p* = 0.020
General health					
Very bad vs. fair	−1.78 (−2.99; − 0.57); *p* = 0.004	0.25 (−12.58; 13.08); *p* = 0.969	−3.95 (−17.80; 9.91); *p* = 0.576	1.55 (−12.40; 15.49); *p* = 0.828	3.11 (−10.54; 16.76); *p* = 0.655
Bad vs. fair	−1.28 (−1.85; − 0.77); *p* < 0.001	−3.19 (−9.22; 2.83); *p* = 0.298	−3.90 (− 10.41; 2.61); *p* = 0.240	−4.82 (−11.37; 1.73); *p* = 0.149	−0.88 (−7.29; 5.53); *p* = 0.787
Good vs. fair	0.70 (0.38; 1.02); *p* < 0.001	8.33 (4.93; 11.72); *p* < 0.001	8.84 (5.18; 12.51); *p* < 0.001	8.12 (4.43; 11.81); *p* < 0.001	7.87 (4.27; 11.48); *p* = < 0.001
Very good vs. fair	1.35 (0.89; 1.81); *p* < 0.001	18.04 (13.17; 22.91); *p* < 0.001	17.61 (12.35; 22.87); *p* < 0.001	18.52 (13.23; 23.82); *p* < 0.001	17.87 (12.68; 23.05); *p* < 0.001
Country					
France vs. Germany	0.32 (−0.11; 0.75); *p* = 0.143	1.27 (− 3.25; 5.79); *p* = 0.580	0.95 (− 3.93; 5.83); *p* = 0.703	0.80 (− 4.11; 5.71); *p* = 0.749	2.02 (− 2.79; 6.83); *p* = 0.410
Netherlands vs. Germany	−0.43 (− 0.84; − 0.03); *p* = 0.035	−3.64 (− 7.91; 0.62); *p* = 0.094	−4.46 (− 9.06; 0.14); *p* = 0.057	− 3.38 (−8.02; 1.25); *p* = 0.152	−3.18 (− 7.71; 1.35); *p* = 0.169
Poland vs. Germany	0.47 (−0.08; 1.01); *p* = 0.092	3.34 (− 2.43; 9.10); *p* = 0.256	1.63 (− 4.60; 7.85); *p* = 0.608	0.31 (−5.95; 6.58); *p* = 0.922	8.02 (1.89; 14.15); *p* = 0.010
Sweden vs. Germany	0.69 (0.22; 1.17); *p* = 0.004	2.68 (− 2.34; 7.71); *p* = 0.295	1.06 (− 4.36; 6.49); *p* = 0.701	2.03 (− 3.43; 7.49); *p* = 0.466	5.02 (− 0.32; 10.37); *p* = 0.066
UK vs. Germany	−0.33 (− 1.02; 0.37); 0.355	3.28 (− 4.06; 10.62); *p* = 0.380	0.57 (− 7.36; 8.50); *p* = 0.888	0.62 (− 7.36; 8.60); *p* = 0.879	8.71 (0.90; 16.52); *p* = 0.029

Data are expressed as the β (95% confidence interval) and *p* value derived from linear regression

There was a consistent association between the center score and satisfaction with care across all five patient-reported indicators (CSQ-4, YHC-SUN total score and three subscales) for both participants with good and participants with very good care health statuses (each compared to fair health status as a reference category), whereas no significant associations could be identified for patients reporting bad or very bad health statuses for any YHC-SUN score except for the general satisfaction with care (Table 4). We found a significant interaction of the center score and the age of the participant on satisfaction with care: in older participants the center score had a higher impact on satisfaction compared to younger participants (*p* < 0.05 for all five indicators). With respect to participating countries, associations between the center score and patient-reported satisfaction were quite different. On the level of general satisfaction (CSQ-4), the association was somewhat smaller for the Netherlands (*p* < 0.05) and higher for Sweden (*p* < 0.005), both compared to “Germany” as the reference category. On the level of satisfaction with specific health care services (YHC-SUN), significant associations between the center score and patient-reported indicator were restricted to the subscale doctor’s behavior indicating higher values for Poland and UK (each *p* < 0.05) compared to Germany.

## Discussion

### Variation of care in DSD conditions

We found large differences between the center scores both within a diagnostic group and within countries. On an individual patient level, the center scores ranged from 0 to 14, meaning that patients with the same diagnosis might have no access or full access to multidisciplinary care. In Germany, with four recruiting centers, we observed remarkable variations across centers and within centers with respect to care for different conditions. In contrast, in France, which also had four recruiting centers, we found much less difference in the center scores between centers and within centers. We assume that the early adoption of strategies to organize care for rare diseases in multidisciplinary centers of reference in France and of more centralized health service policies may have contributed to less regional variations and higher levels of multidisciplinary care in general. For rare diseases in France, the services have been organized in 131 centers of reference and 501 centers of competence, while in Germany, there is an unknown number of self-defined centers of excellence and networks. The process of certification, including quality criteria of care, is ongoing. Furthermore, the research approach in France appears to be more directive compared to Germany [29, 30]. In the two Dutch centers, we recognize a portrait of high levels of specialization for one condition within the diagnostic groups. This may indicate a high level of specialization for those conditions. Some policy recommendations suggest that all rare conditions within one larger diagnosis-related group such as “all endocrine disorders” should be allocated to one center [5].

### Satisfaction with health care services

Differences in satisfaction with care were evident across the diagnostic groups with the highest levels of satisfaction among women with CAH, including the highest scores on the subscales of satisfaction with information about the diagnosis, patient-centered care and doctors’ behaviors. This confirms results from the German Network study on the satisfaction with care in adults with DSD (excluding TS and KS) that showed that women with congenital adrenal hyperplasia reported the highest scores for satisfaction with care [31]. The authors interpreted this with specific characteristics of the condition: early identification (in countries with neonatal screening programs), evidence-based guidelines, accessible hormone treatment, larger caseloads in specialized clinics, and frequent organized transition to adult care. In TS, some characteristics are similar (i.e., access to guidelines for treatment), but the complexity of the condition and failures during the transitional phase to adult care may lead to lower levels of satisfaction [32]. Both males and females with XY-DSD reported low levels of overall and specific satisfaction with care, which is consistent with previous findings and with the fact that this condition group encompasses extremely rare conditions, a high rate of conditions undiagnosed on the molecular level, late diagnoses and high levels of stigmatization and secrecy [31]. The level of satisfaction with care was low in both male groups: XY-DSD and KS. Male XY-DSD participants may have experienced the most extensive medical and surgical procedures and multiple encounters with the health care system. For participants with KS, we have no evidence from the literature for low satisfaction with care, and we will perform a more in-depth analysis to explore the finding in a subsequent study.

### Association between structural quality and satisfaction with care

Overall association of the center score for structural quality of care with individual participant satisfaction with care was significant, highlighting the appreciation of the participants in having access to multidisciplinary care. Our results show that the patients perceive a good subjective result quality leading to their satisfaction if certain services exist. Consequently, a higher (objective) structural quality leads to a higher (subjective) result quality. Even though this association does not vary that much across different diagnostic groups, the disease burden in terms of health status has an impact on the relationship between structural and result quality: healthier patients substantially benefit more from structural investments. The finding requires more in-depth analyses and qualitative research methods to determine the reasons why patients with poor health benefit less from the infrastructure provided as they appear to be an especially vulnerable population and would appear to require more integrated and multifaceted care compared to those with better health.

In older versus younger participants the center scores indicating structural quality have a stronger effect on satisfaction with care in all measures. We speculate that longer exposure to the care in a given clinic may have contributed to participants’ experiences and opinion formation as well as perceptions of needs.

This relation allows us to assume that the process quality is sufficient to transfer the investments into structural quality and service quality, i.e., it is an indication that the centers with a high structural quality are working quite well. However, a high structural quality does not allow us to conclude that this has an impact on the quality of life of the patient. This must be assessed separately and is beyond the scope of this paper. Analyses of health care needs in the specific forms of DSD in more detail are under way.

### Limitations

The response rate of this study was about 30% and the results might not be representative for the population with DSD. However, the response rate was calculated from the number of patients being cared for in the collaborating centers now and received an invitation per mail, telephone or personal contacts. Many former patients could not be traced. The response rate for those contacted directly by their physician in the clinic was 76% [21] and we therefore believe that the results are representative for patients who contemporarily receive their care in the respective clinics.

This paper does not provide information on individual participant’s personal experiences with the care they received in the center. The collected data regarding utilization of care in individual patients in dsd-LIFE was largely related to past medical care in childhood and adolescence and not specifically linked to a site of care. Thus, we are unable to examine personal experiences of care and reported unmet needs as a moderator between the association of structural quality of care and participants’ individual satisfaction with care. The new established measure coined “center score” is self-constructed based on a conceptual framework and has not been validated in other studies. We deleted three items concerning inclusion of all age groups, provision of expert advice / second opinions and teaching and training activities because they had high ceiling effects in this sample of specialized tertiary care centers- they may, however, be instructive in other settings.

As widely known for satisfaction scores, this type of assessment shows ceiling effects. Nevertheless, variability of the scores is sufficient, as indicated by variations of the score on the level of single patients or individual centres. Thus, a lack of variability of satisfaction scores (especially as plotted against the center scores) might indicate that satisfaction with health care services in general is on a high level and does not primarily depend on the structural quality of the centres but also on the processes and outcomes it facilitates.

## Conclusion

Further studies are needed to assess the impact of care in centers of reference/excellence or competence not only on patient satisfaction with care but also on patient health outcomes, health care budgetary investments in such centers and investment returns in terms of both quality of care and quality of life. Comparative effectiveness research across Europe may lead to more insight into beneficial structures and processes and the overall strategy of care for people with rare diseases. However, to decrease variability, the development and implementation of standards of care that are based on evidence from sufficiently large controlled clinical trials is crucial for harmonization of care across Europe.
